# Ischemia and reperfusion injury following cardioplegic arrest is attenuated by age and testosterone deficiency in male but not female mice

**DOI:** 10.1186/s13293-019-0256-4

**Published:** 2019-08-23

**Authors:** Anjali Ghimire, Elise S. Bisset, Susan E. Howlett

**Affiliations:** 1 Department of Pharmacology, Dalhousie University, Sir Charles Tupper Medical Building, 5850 College Street, PO Box 15000, Halifax, Nova Scotia B3H 4R2 Canada; 2 Department of Medicine (Geriatric Medicine), Dalhousie University, Halifax, Nova Scotia Canada

**Keywords:** Ischemia/reperfusion injury, Cardiac surgery, Cardioplegia, Cardioprotection, Sex differences, Sex hormones, Aging, Cardiac contraction

## Abstract

**Background:**

Cardiovascular disease increases with age in both sexes. Treatment can require cardiac surgery, where the hearts are pre-treated with protective cardioplegic solution before ischemia and reperfusion (I/R). While endogenous estrogen is beneficial in I/R, whether testosterone is involved is uncertain and whether age modifies responses to I/R is unclear. We investigated sex- and age-specific differences in I/R injury in the hearts pre-treated with clinically relevant cardioplegic solution.

**Methods:**

The hearts were isolated from young (6–9 months) and old (20–28 months) mice of both sexes and perfused (Langendorff) with Krebs-Henseleit buffer (15 min, 37 °C), followed by St. Thomas’ two cardioplegia (6 min, 6–7 °C), global ischemia (90 min, 23–24 °C), and reperfusion (30 min, 37 °C). The hearts were perfused with triphenyltetrazolium chloride to quantify infarct area. Testosterone’s role was investigated in gonadectomized (GDX, 6–9 months) male mice; serum testosterone and estradiol were measured with ELISA assays.

**Results:**

Left ventricular developed pressure (LVDP) recovered to 67.3 ± 7.4% in the old compared to 21.8 ± 9.2% in the young male hearts (*p* < 0.05). Similar results were seen for rates of pressure development (+dP/dt) and decay (−dP/dt). Infarct areas were smaller in the old male hearts (16.6 ± 1.6%) than in the younger hearts (55.8 ± 1.2%, *p* < 0.05). By contrast, the hearts from young and old females exhibited a similar post-ischemic functional recovery and no age-dependent difference in infarcts. There was a sex difference in the young group, where ventricular function (LVDP, +dP/dt, −dP/dt) recovered better and infarcts were smaller in females than males. Estradiol levels were highest in young females. Testosterone was high in young males but low in females and old males, which suggested beneficial effects of low testosterone. Indeed, the hearts from GDX males exhibited much better recovery of LVDP in reperfusion than that from intact males (values were 64.4 ± 7.5 % vs. 21.8 ± 9.2%; *p* < 0.05). The GDX hearts also had smaller infarcts than the hearts from intact males (*p* < 0.05).

**Conclusions:**

Although age had no effect on susceptibility to I/R injury after cardioplegic arrest in females, it actually protected against injury in older males. Our findings indicate that low testosterone may be protective against I/R injury following cardioplegic arrest in older males.

**Electronic supplementary material:**

The online version of this article (10.1186/s13293-019-0256-4) contains supplementary material, which is available to authorized users.

## Background

Cardiovascular disease (CVD) is a leading cause of death globally, and its prevalence increases with age in both sexes [1]. However, women typically develop CVD 10 to 15 years later than men [2]. It is believed that endogenous estrogen is cardioprotective in women [3], and indeed, the risk for CVD rises markedly after menopause [4]. Many CVDs, including coronary artery disease and valvular heart disease, require surgical intervention as a part of treatment. During cardiac surgery, the heart is susceptible to ischemia/reperfusion (I/R) injury [5, 6]. Preclinical studies have shown better recovery of contractile function in the young adult female hearts exposed to global ischemia when compared to age-matched males [7–9]. This has been largely explained by protective effects of estrogen. However, it is clear that testosterone levels also decline with age in males starting in the third decade [10]. There is growing evidence that low testosterone levels predispose toward CVDs [11], but the role of testosterone in the recovery of the heart after ischemic insult is not well understood.

Age itself causes distinct patterns of structural and functional remodeling of the heart in both sexes [12–17]. These age-dependent changes may increase the susceptibility of older adults to I/R injury in various settings, including cardiac surgery. Indeed, some studies have shown higher rates of adverse outcomes following cardiac surgery in older individuals, especially older women (≥ 55 years) [18, 19]. By contrast, others have reported that morbidity and mortality are similar in older adults (e.g., ≥ 65 years) of both sexes following coronary artery bypass graft surgery [20, 21]. Interestingly, Filsoufi and colleagues [22] showed a minimal increase in postoperative morbidity and mortality in older patients (≥ 80 years) compared to younger patients (70 to 79 years) of both sexes. Therefore, whether age itself increases the susceptibility to ischemic injury in the setting of cardiac surgery, and whether this differs between the sexes, is unclear.

In cardiac surgery, the heart is arrested with a “cardioplegic solution” (also known as cardioplegia). Cardioplegic solutions are designed to rapidly inhibit contractions, reduce metabolic rate, and decrease O_2_ demand by the myocardium [23, 24]. These actions prevent the heart from beating to facilitate cardiac surgery and protect the heart from I/R injury [25]. Although the use of cardioplegic solutions has improved outcomes following cardiac surgery [25], different levels of protection are reported in specific populations. For example, better outcomes after cardiac surgery are observed when del Nido cardioplegia is used when compared to standard cardioplegia, in particular in pediatric patients [26, 27]. There is also evidence for sex-specific differences in the efficacy of standard cardioplegic solution, with lower cardiac protection reported in females compared to males [28]. These studies suggest that the ability of cardioplegic solutions to protect the heart may vary with both age and sex.

In this study, we investigated age- and sex-specific differences in recovery of cardiac contractile function following exposure to hypothermic St. Thomas’ Hospital cardioplegic solution No. 2 (STH2); STH2 is a crystalloid cardioplegic solution that is widely used in clinical practice [29, 30]. Our objectives were to determine sex- and age-specific differences in the functional recovery of the Langendorff-perfused hearts treated with hypothermic STH2 cardioplegia and to explore links between serum testosterone levels and the ability of the heart to recover from cardioplegic arrest.

## Methods

### Experimental animals

Experimental protocols were approved by the Dalhousie Committee on Laboratory Animals and followed the guidelines provided by the Canadian Council on Animal Care (CCAC, Ottawa, ON: Vol 1, 2nd edition, 1993; revised March 2017). Studies were reported according to the Animal Research: Reporting of In Vivo Experiments (ARRIVE) guidelines [31]. Male, female, and gonadectomized (GDX; operation at 1 month) male C57BL/6 mice were obtained either from Charles River Laboratories (St. Constant, QC, Canada) or The Jackson Laboratory (Bar Harbor, ME, USA). All mice were housed in micro-isolator cages in the Carleton Animal Care Animal Facility at Dalhousie University. They were aged in the animal care facility and exposed to a 12-h light/dark cycle. Food and water were provided ad libitum. Five groups of mice were used in our studies: young adult males and females (6 to 9 months), old adult males and females (20 to 28 months), and young adult GDX males (6 to 9 months).

### Langendorff-perfused mouse heart model of cardioplegia

**Fig. 1:**
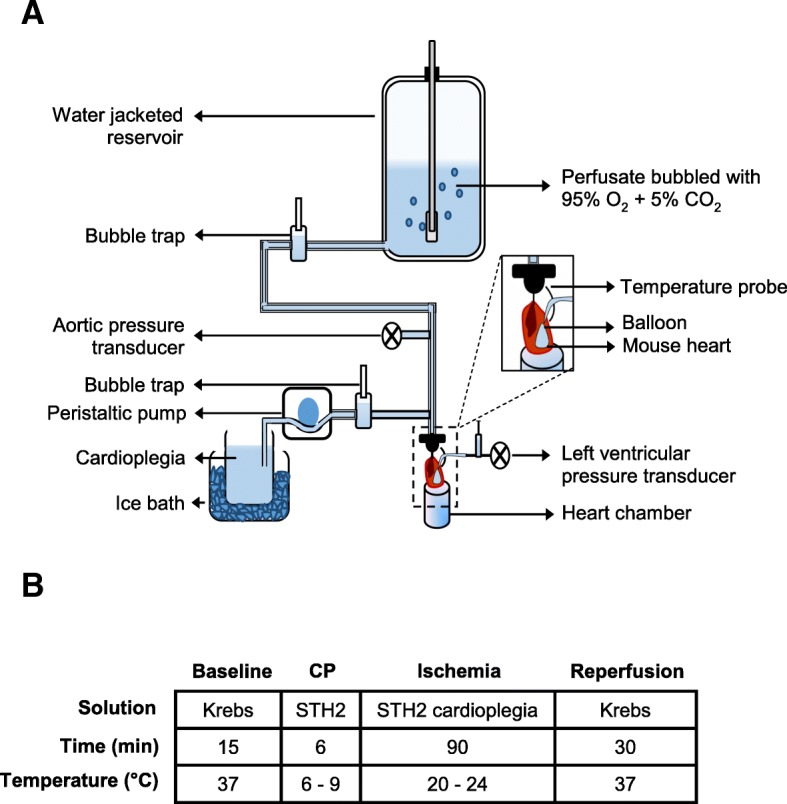
Experimental setup and protocol. **a** Schematic diagram of the modified Langendorff retrograde heart perfusion system. **b** The experimental timeline. Baseline perfusion of the mouse heart was done with Krebs-Henseleit buffer solution at 37 °C (15 min). Then, the perfusion was switched to St Thomas’ II cardioplegia (6–9 °C) for 6 min. Following cardioplegic perfusion, the heart was subjected to 90 min of ischemia, where the heart remained submerged in cardioplegia at room temperature (20–24 °C). Subsequently, the heart was reperfused with Krebs-Henseleit solution (37 °C) for 30 min and recovery of heart function was recorded

In this study, the traditional Langendorff-perfusion system was modified to mimic the clinical delivery of cardioplegia, as shown in Fig. 1a. Specifically, a second reservoir containing cardioplegic solution was added and this solution was kept on ice. In addition, the lines that delivered cold cardioplegia to the heart were encased in tubing with circulating ice-cold water. This ensured that cardioplegia was delivered to the heart at between 6 and 9 °C, as used in clinical studies [32], and this was verified with a temperature probe (Fig. 1a).

Mice were weighed then anesthetized with sodium pentobarbital (220 mg/kg IP) co-administered with heparin (3000 U/kg) to prevent coagulation. A longitudinal chest incision was made to expose the heart and a suture was loosely tied around the aorta. The aorta was then cut, rapidly cannulated, secured, and perfused with 37 °C Krebs-Henseleit buffer solution (in mM): 108.3 NaCl, 4.7 KCl, 25 NaHCO_3_, 1.2 MgSO_4_, 1.2 KH_2_PO_4_, 11 glucose, 0.79 Na-pyruvate, and 1.8 CaCl_2_, equilibrated with 95% O_2_ and 5% CO_2_ (pH 7.4). The cannulated heart was then mounted for Langendorff perfusion at a constant pressure of 80 ± 1 mmHg, as shown in Fig. 1a. A custom-made balloon was inserted in the left ventricle and inflated with degassed distilled water to yield a minimum left ventricular pressure of 10.2 ± 0.5 mmHg. Tibia length was measured to provide an estimate of body size for data normalization.

The experimental protocol is illustrated in Fig. 1b. The heart was perfused with Krebs-Henseleit buffer for 15 min and baseline measurements of left ventricular developed pressure (LVDP), heart rate, coronary flow rate, rate of pressure development (+dP/dt), and rate of pressure decay (−dP/dt) were recorded. Then, the heart was perfused with hypothermic (6–9 °C) STH2 cardioplegia (in mM): 110 NaCl, 10 NaHCO_3,_ 16 KCl, 16 MgCl_2_, and 1.2 CaCl_2_ (pH 7.8), which arrested the heart immediately. After 6 min of cardioplegia, the heart was subjected to 90 min of global ischemia by stopping the flow of solution and submerging the heart in room temperature (23–24 °C) cardioplegia as done clinically [33]. The heart was reperfused with Krebs-Henseleit buffer for 30 min, and functional parameters were evaluated to determine the extent of recovery.

### Measurement of infarct area

Infarct sizes following cardioplegic arrest and reperfusion were measured by staining the hearts with triphenyltetrazolium chloride (TTC) (Sigma-Aldrich, Oakville, ON, Canada). Following reperfusion, the cannulated heart was removed from the Langendorff apparatus and perfused with 1% TTC solution (0.1 g TTC in 10 ml Krebs-Henseleit buffer solution) delivered via a 10 ml syringe. Then, the heart was incubated for 45 min at 37 °C in TTC solution and then weighed. Following incubation, the heart was fixed in 10% formalin (Sigma-Aldrich, Oakville, ON, Canada) for at least 48 h. Then, the heart was evenly sliced (1 mm slices) with a heart slicer matrix (Zivic Instruments, Pittsburgh, PA, USA). Photographs of the heart slices were used to quantify infarct area with computerized planimetry (Adobe Photoshop 8 CS, Adobe System Incorporated, USA) and ImageJ 1.50i (National Institutes of Health, USA). The pale pink/white areas in the heart slices were scored as the infarcted regions while the deep red areas were scored as viable tissue. Total infarct area was calculated and expressed as a percentage of the total heart area. Each experimental group was assigned a unique code and the data were analyzed blinded.

### Steroid hormone measurement

A blood sample was collected either by facial vein puncture or from the aorta during cannulation and allowed to clot at room temperature for 30 min. Samples were then centrifuged at 1500×*g* for 10 min at − 4 °C. The serum (supernatant) was used to assay the levels of testosterone and estradiol with a mouse testosterone ELISA kit (Crystal Chem Inc., Elk Grove Village, IL) or a rat estradiol ELISA kit (Crystal Chem Inc., Elk Grove Village, IL), following the manufacturer’s suggested procedures. Testosterone and estradiol concentrations were determined from a four-parameter logistic curve fit, as recommended in the instructions. Values that were below the limit of detection of the assay were estimated from the limit of quantification divided by the square root of two [34].

### Data analysis and statistics

Functional parameters were evaluated to determine if contractile function recovered back to baseline levels after cardioplegic arrest. Therefore, we normalized the data for each heart to its own baseline values to control for potential differences in basal state between the hearts. This allowed us to determine whether each heart recovered back to its starting levels or if recovery of function was impaired in any particular group. Functional parameters were calculated as follows. LVDP was calculated as the height of developed pressure (systolic pressure - end-diastolic pressure). The steepest slope during the upstroke for left ventricular pressure recordings was quantified as +dP/dt and the slope during the downstroke of pressure recordings provided a measure of −dP/dt. The left ventricular performance was also assessed by calculating the rate pressure product (RPP) to correct for heart rate and heart size. The RPP = (LVDP × heart rate)/heart weight.

Data were analyzed with either SigmaPlot 11.0 (Systat Software, Inc., Point Richmond, CA, USA) or IBM SPSS Statistics 25. Comparisons between groups for morphometric data, infarct area, coronary flow rate, contracture, and testosterone levels were conducted using two-way ANOVA, with age and sex as main factors; a Holm-Sidak post hoc test was used. Differences between groups for the functional parameters (LVDP, +dP/dt, −dP/dt, and RPP) were evaluated with a mixed three-way ANOVA with two between factors (age, sex) and one within factor (time); a Bonferroni post hoc test was used. Functional differences between the hearts from intact and GDX mice were assessed with a mixed two-way repeated measures ANOVA, with GDX and time as main factors; the Holm-Sidak post hoc test was used. We used a parametric test (Student’s *t* test) to evaluate the effect of GDX on coronary flow because these data were normally distributed. When data were not normally distributed (e.g., infarct area and coronary flow), we used the non-parametric Mann-Whitney *U* test. All the data are presented as the mean ± SEM; differences are reported as significant if *p* < 0.05. Data were plotted with SigmaPlot 11.0.

## Results

### Development of a murine heart model of cardioplegia

**Fig. 2:**
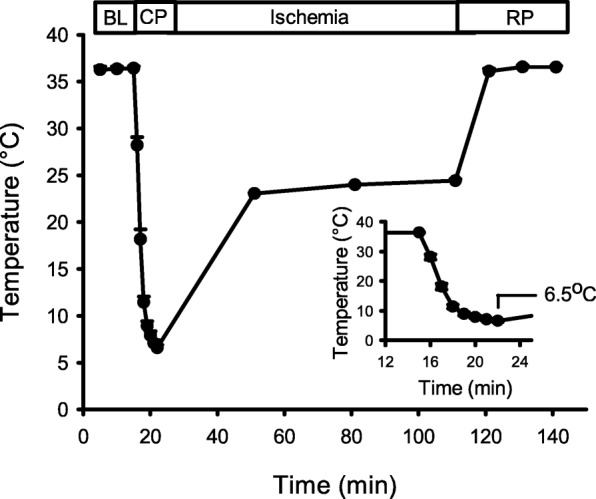
Temperature regulation throughout the experiment. During 15 min of baseline (BL) perfusion the temperature of the myocardium was maintained at approximately 37 °C. Following 6 min of perfusion with hypothermic cardioplegia (CP), the temperature dropped to between 6 and 9 °C. Next, the heart was submerged in cardioplegia at room temperature and temperature was maintained between 20 and 24 °C. Finally, the heart was reperfused (RP) with Krebs-Henseleit buffer and the temperature recovered to 37 °C. The inset graph is an enlarged view of the temperature drop during cardioplegic perfusion. Values represent the mean ± SEM for 15 experiments

To develop a clinically relevant mouse model of cardioplegia, we modified a standard Langendorff perfusion system as described in the methods. These modifications allowed us to control the temperature in the ex vivo mouse heart to mimic conditions experienced during cardiac surgery. As shown in Fig. 2, baseline myocardial temperature was maintained at physiological levels (36.3 ± 0.2 °C). The hearts were then perfused with hypothermic STH2 cardioplegia for 6 min, which rapidly reduced myocardial temperature to 6.5 ± 0.3 °C by the end of cardioplegia (Fig. 2, inset). Next, the hearts were subjected to 90 min of global ischemia at room temperature (23–24 °C), and the myocardial temperature rose to 6.5 ± 0.3 °C by the end of ischemia. The hearts were then reperfused for 30 min at physiological temperature (36.4 ± 0.1 °C at the end of reperfusion). This protocol provided a reproducible mouse model of cardioplegia with temperatures very similar to those experienced during cardiac surgery.

### Infarcts were smaller in the hearts from old males compared to young males, while infarct sizes were not affected by age in females

**Fig. 3:**
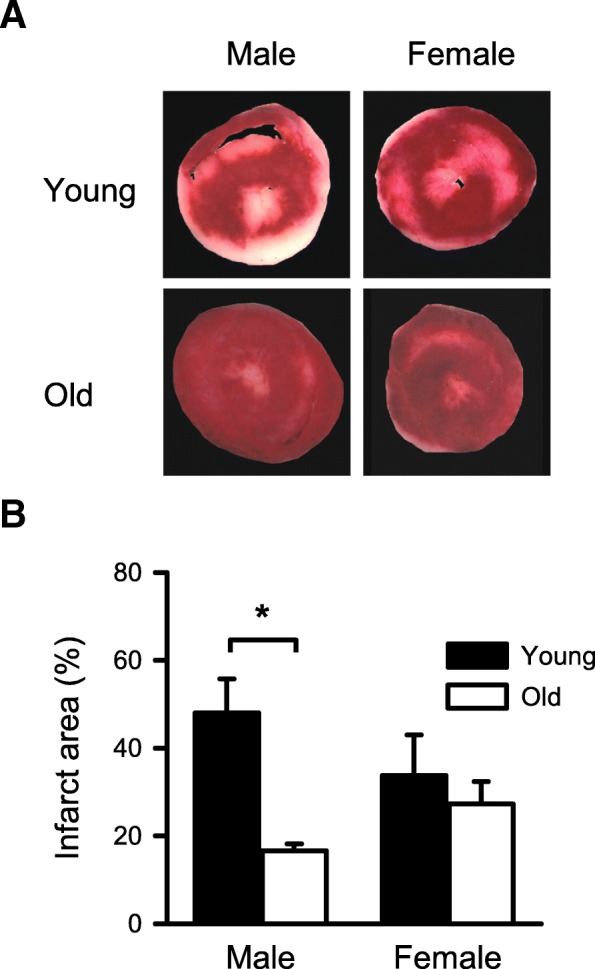
Comparison of the infarct area in the hearts from young and old mice of both sexes. **a** Representative heart sections from young male (top-left), old male (bottom-left), young female (top-right), and old female (bottom-right) mice; the hearts were stained with TTC. **b** The old male mouse hearts had significantly smaller infarcts when compared to the young male hearts. However, the hearts from female mice had similar infarct areas, regardless of age. Values are expressed as the mean ± SEM. Data were analyzed with two-way ANOVA, with age and sex as the main factors. The overall effect of age was statistically significant. The asterisk denotes significantly different from young male (*p* < 0.05), and the number sign denotes significantly different from young female (*p* < 0.05); the Holm-Sidak post hoc test was used. Detailed statistical analysis is presented in Additional file 1: Table S1. Young male, *n* = 4; old male, *n* = 6; young female, *n* = 4; old female, *n* = 5

We next used this model to investigate age- and sex-related differences in the extent of myocardial injury in the hearts treated with STH2 cardioplegia prior to I/R. The percentage of infarct area was compared between the young and old mouse hearts of both sexes with a two-way ANOVA, with age and sex as main factors; detailed statistical analysis is presented in Additional file 1: Table S1. Figure 3a shows representative photographs of heart slices from each group. The percent infarct area was significantly lower in the hearts from old male mice when compared to young males (Fig. 3b). However, there was no difference in infarct area between the young and old hearts from female mice (Fig. 3b). These data indicate that the hearts from older males had a less myocardial injury when compared to younger males. There was also a sex-specific effect such that young males had much larger infarcts than young females (Fig. 3b).

### Contractile function recovered better in old compared to the young male hearts, while recovery in females was similar regardless of age

**Fig. 4:**
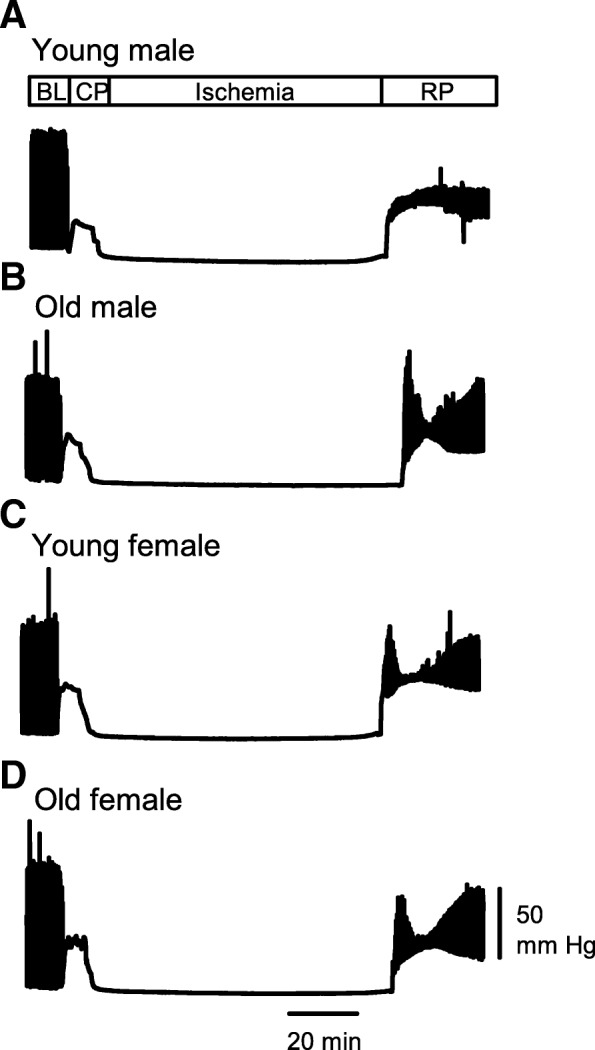
Representative experimental recordings. Sample left ventricular pressure recordings illustrate responses during baseline (BL), cardioplegic perfusion (CP), ischemia, and reperfusion (RP). The baseline section showed normal cardiac function during initial perfusion of the heart. When the heart was perfused with hypothermic STH2 cardioplegia, all the contractile activity ceased and a rapid cooling contracture was seen. During ischemia, the heart relaxed. Contractile activity recovered to varying extent in reperfusion. **a** Young male. **b** Old male. **c** Young female. **d** Old female

We next used this model to investigate age- and sex-related differences in the recovery of contractile function following exposure to cardioplegia, then I/R. Figure 4 shows representative pressure recordings of contractile function throughout an experiment from young male (Fig. 4a), old male (Fig. 4b), young female (Fig. 4c), and old female (Fig. 4d) mice. These recordings show left ventricular pressure at baseline, during cardioplegia, in ischemia, and during reperfusion. In all groups, exposure to cardioplegia abolished contractions and caused an increase in baseline pressure, known as a rapid cooling contracture [35]. Subsequent exposure to ischemia inhibited contractions and abolished the contracture in all groups. Reperfusion was accompanied by a baseline contracture, but it also initiated recovery of contractile function that was less complete in the young male heart when compared to either the older male or females at any age. There were no age- or sex-dependent differences in the pressure responses during cardioplegia or ischemia (data not shown). However, the degree of recovery of contractile function varied between groups in reperfusion, so this was explored in detail.

**Fig. 5:**
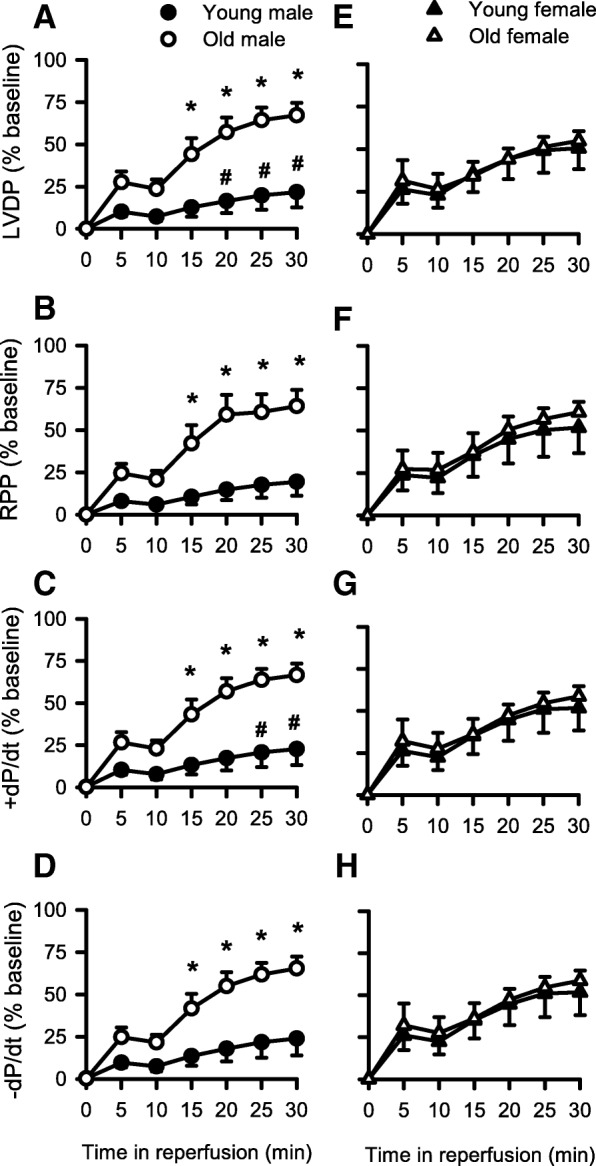
Sex- and age-specific differences in the recovery of contractile function during reperfusion. All data were normalized to the baseline values. **a** LVDP recovered significantly better in the hearts from old males compared to young males in reperfusion. There was also a significant effect of sex in the young adult group, where young females recovered significantly better than young males. The recovery of **b** RPP, **c** +dP/dt, and **d** −dP/dt in the old male hearts was also significantly better than in the young male hearts, although the effect of sex was only significant in late reperfusion for +dP/dt. By contrast, **e** LVDP, **f** RPP, **g** +dP/dt, and **h** −dP/dt recovered to the same extent in the female hearts, regardless of age. Values are expressed as the mean ± SEM. Results were analyzed with a three-way mixed ANOVA with two between factors (age, sex) and one within factor (time). The asterisk denotes significantly different from young male (*p* < 0.05); the number sign denotes significantly different from young female (*p* < 0.05). Detailed statistical analysis is presented in Additional file 2: Table S2. Young male, *n* = 5; old male, *n* = 6; young female, *n* = 6; old female, *n* = 5

Figure 5 shows recovery of LVDP, RPP, +dP/dt, and −dP/dt in reperfusion in the hearts isolated from young and old mice of both sexes. The values in reperfusion were normalized to baseline values and were then expressed as the percentage of baseline. We first explored the effect of age and sex on recovery of contractile function with a three-way mixed ANOVA with two between factors (age, sex) and one within factor (time); detailed statistical analysis is presented in Additional file 2: Table S2. Mean data show that the recovery of LVDP was substantially better in the old male hearts than in the young male hearts, and this was significant at 15 to 30 min of reperfusion (Fig. 5a). As both heart weight and heart weight to tibia length ratios (indicative of cardiac hypertrophy) increased with age in males but not females (Table 1; Additional file 1: Table S1), we normalized LVDP by heart weight and heart rate by calculating the RPP. Results showed that RPP recovered significantly better in reperfusion in old males compared to young males (Fig. 5b). Recovery of +dP/dt and −dP/dt was also better in the old male hearts when compared to younger males (Fig. 5c, d). By contrast, contractile function recovered to the same extent in the hearts from female mice, regardless of their age (Fig. 5e–h). We also found that there was a significant effect of sex in the young group, where recovery of LVDP (Fig. 5a, e) and +dP/dt (Fig. 5c, g) was worse in young males than in young females. Taken together, these results show that the young male mouse hearts had much less complete recovery of function in reperfusion when compared to the older male hearts and to the hearts from young females. Thus, only the male mouse hearts showed age-specific differences in the recovery of contractile function after treatment with STH2 cardioplegia prior to I/R. This more complete recovery of contractile function observed in the older male hearts may be explained, at least in part, by smaller infarcts when compared to younger males (Fig. 3).

**Table 1:** Morphometric data for mice used in this study

Parameter*	Young male	Old male	Young female	Old female
Body weight (g)	33.8 ± 2.8	35.1 ± 1.6	30.8 ± 1.9	37.3 ± 3.5
HW (mg)	217.7 ± 12.9	264.2 ± 25.5	191.7 ± 16.4	189.2 ± 4.0^a^
HW:BW (mg/g)	6.6 ± 0.4	7.7 ± 0.9	6.4 ± 0.7	5.2 ± 0.4
TL (mm)	18.6 ± 0.1	18.4 ± 0.4	18.7 ± 0.05	19.0 ± 0.2
HW:TL (mg/mm)	11.7 ± 0.7	14.4 ± 1.3^b^	10.2 ± 0.9	9.6 ± 0.2^a^
Number of mice	6	6	6	5

*HW* heart weight, *BW* body weight, *TL* tibia length

*Numbers represent mean ± SEM. Data were analyzed with two-way ANOVA with age and sex as main factors. Detailed statistical analysis is presented in Additional file 1: Table S1

^a^Denotes significant effect of sex (*p* < 0.05)

^b^Denotes significant effect of age (*p* < 0.05)

### Coronary flow rate and contracture in reperfusion were similar regardless of age or sex

**Fig. 6:**
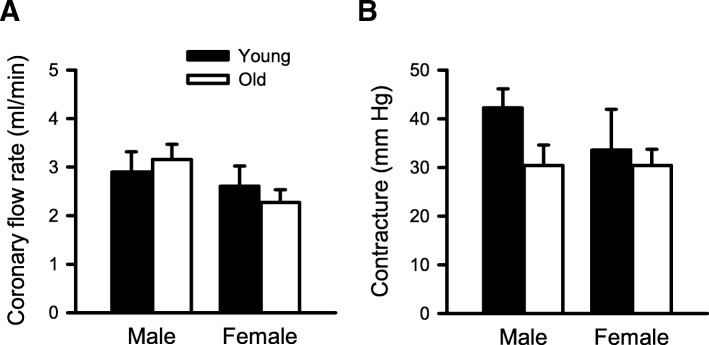
Coronary flow rates and contracture levels were similar in reperfusion regardless of age or sex. **a** There was no difference in the coronary flow rate in reperfusion between any of the experimental groups. **b** Contracture levels were also similar in the young and old male and female mouse hearts in reperfusion. Values are expressed as the mean ± SEM. Results were analyzed with two-way ANOVA, with age and sex as main the factors. Young male, *n* = 5; old male, *n* = 6; young female, *n* = 6; old female, *n* = 5

As differences in coronary flow rates could affect the recovery of contractile function in reperfusion, we compared the rates of myocardial perfusion in reperfusion in all four groups with a two-way ANOVA (age and sex as main factors). When coronary flow rates were quantified at the end of reperfusion (Fig. 6a), we found that there were no age- or sex-related differences. We compared the magnitude of reperfusion contracture as an index of myocardial damage and found that the level of contracture in reperfusion was similar in young and old mice of both sexes as shown in Fig. 6b. These results show that coronary flow rates and reperfusion contractures were similar in all four groups and suggest that differences in the extent of myocardial perfusion do not account for improved recovery of function in older males.

### Low serum testosterone levels contribute to improved recovery of contractile function and smaller infarct sizes in reperfusion

**Fig. 7:**
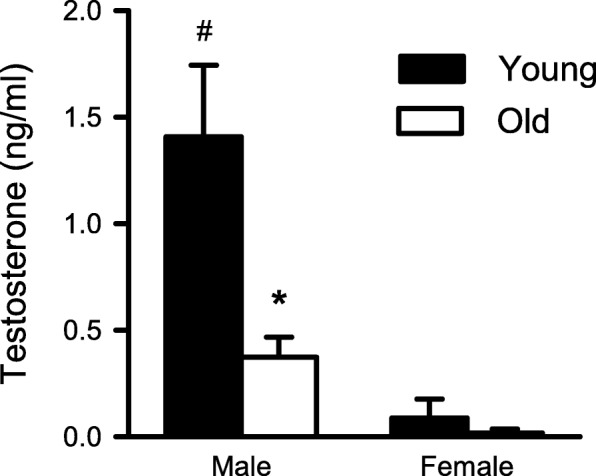
Comparison of serum testosterone levels in young and old mice of both sexes. Serum testosterone concentrations were significantly lower in old males compared to young males. Testosterone levels were low in females regardless of age. Data were analyzed with two-way ANOVA, with age and sex as main factors. Values are expressed as the mean ± SEM. The asterisk denotes significantly different from young male (*p* < 0.05) and the number sign denotes significantly different from young female (*p* < 0.05); the Holm-Sidak post hoc test was used. Detailed statistical analysis is presented in Additional file 1: Table S1. Young male, *n* = 3; old male, *n* = 4; young female, *n* = 3; old female, *n* = 5

Results presented so far indicate that younger males had larger infarcts and less complete recovery of function in reperfusion when compared to older male mice and females at any age. To investigate potential underlying mechanisms, we compared serum testosterone levels in young and older mice of both sexes with a two-way ANOVA with age and sex as main factors; detailed statistical analysis is presented in Additional file 1: Table S1. As shown in Fig. 7a, serum testosterone concentrations in old males were significantly lower than levels in young males. As expected, serum testosterone levels were low in female mice regardless of age (Fig. 7). Interestingly, we again saw a sex-specific effect in the younger group, where testosterone levels were significantly higher in young males than in young females (Fig. 7). To determine whether age and sex differences in estradiol could account for differences in recovery between groups, we also quantified serum estradiol levels. Results showed that estradiol levels were high in young females (9.0 ± 4.8 pg/ml; *n* = 8) but below the level of detection for the assay in older females (*n* = 6). Values for young males also were below the limit of detection (*n* = 6), but estradiol was detectable in older males (6.8 ± 2.2 pg/ml; *n* = 8). Taken together, these data are consistent with the idea that low serum testosterone levels as well as higher estradiol levels may contribute to better functional recovery in reperfusion following cardioplegic arrest in the older male hearts.

**Fig. 8:**
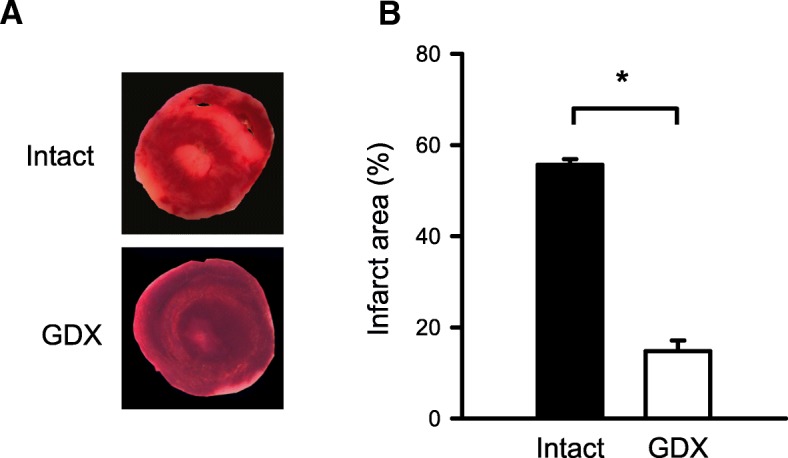
GDX young male mouse hearts had smaller infarcts than controls. **a** Representative heart sections stained with TTC from intact (top) and GDX (bottom) male mice. **b** GDX mouse hearts had significantly smaller infarcts than intact, age-matched controls. Values are expressed as the mean ± SEM. Data were analyzed with a Mann-Whitney *U* test. Results showed that infarcts in intact male hearts were significantly larger than in the hearts from GDX males (*U* = 1.0, *p* = 0.032). The asterisk denotes significantly different from intact male (*p* < 0.05). Intact male, *n* = 5; GDX male, *n* = 4

To test this idea, responses to cardioplegic arrest followed by reperfusion were compared in the hearts from young adult mice that had either intact gonads or a GDX at 1 month of age. GDX dramatically reduced serum testosterone and estradiol so that levels were below the limits of detection for the assays. We then estimated infarct size following cardioplegic arrest and reperfusion by measuring the infarct areas in heart slices from GDX and intact male mice. Figure 8a shows representative photographs of infarcts in the hearts from GDX and intact male mice. Data were analyzed with a Mann-Whitney *U* test. Infarct area was smaller in the GDX hearts than in the hearts from intact mice of the same age (Fig. 8b). Scores in intact males were significantly higher than in GDX males (*U* = 1.0, *p* = 0.032). These observations demonstrate that young mice with low circulating testosterone levels exhibited less myocardial injury after cardioplegia and I/R than young mice with normal testosterone levels.

**Fig. 9:**
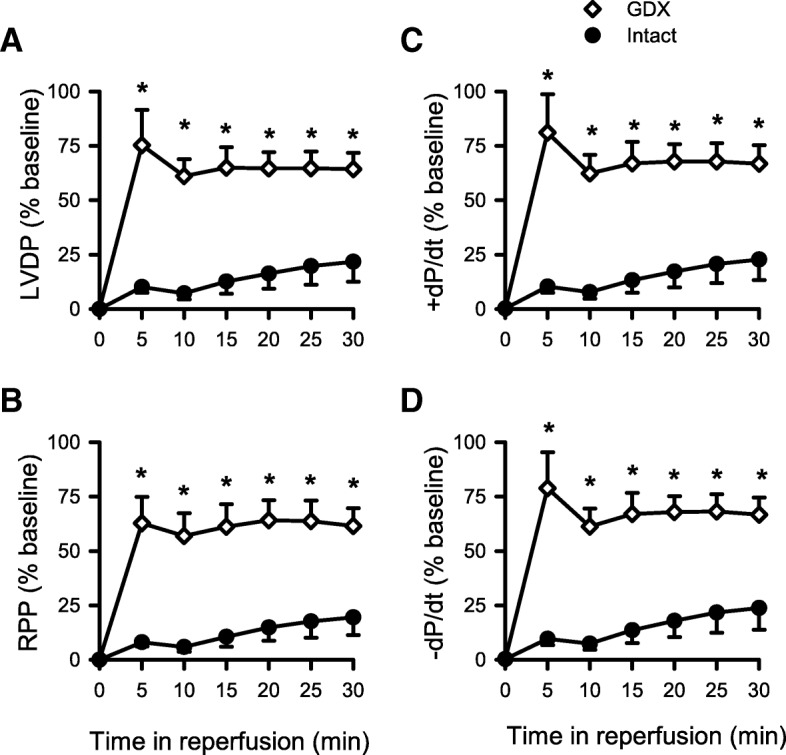
Differences in the recovery of contractile function between GDX and age-matched control male mouse hearts. **a** LVDP recovered significantly better throughout the reperfusion duration in GDX compared to control young male mouse hearts. Similarly, **b** RPP, **c** +dP/dt, and **d** −dP/dt also recovered significantly better in reperfusion in GDX compared to controls. Values are expressed as the mean ± SEM. Data were analyzed with two-way repeated measures ANOVA with GDX as a main factor and time as the repeated measure. Detailed statistical analysis is presented in Additional file 3: Table S3. The asterisk denotes significantly different from intact male mice. Intact male, *n* = 5; GDX male, *n* = 4

**Fig. 10:**
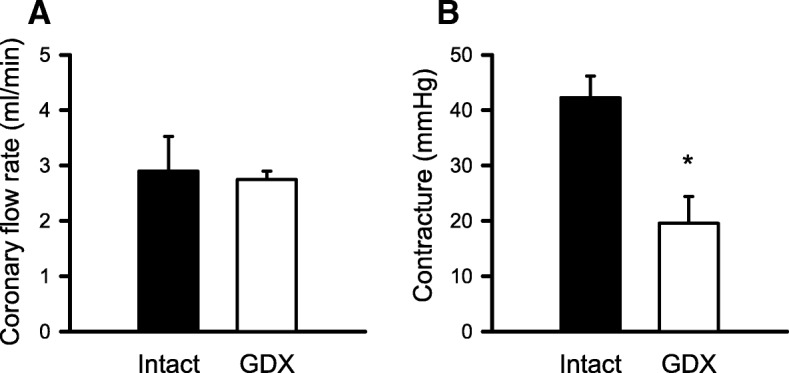
Coronary flow rates were similar in intact and GDX hearts in reperfusion, although GDX reduced the magnitude of contractures seen in reperfusion. **a** Coronary flow data were analyzed with a Mann-Whitney *U* test. Results showed that there was no difference in coronary flow rates in reperfusion between intact and GDX groups (*U* = 7.0, *p* = 0.556). **b** Cardiac contracture levels were evaluated with a *t* test. Results showed that contractures in reperfusion were smaller in the GDX hearts (*t* = 3.686, *p* = 0.008). Values are expressed as the mean ± SEM. Intact male, *n* = 5; GDX male, *n* = 4

Next, we performed functional studies where we exposed the Langendorff-perfused hearts from intact and GDX mice to cardioplegia followed by I/R. Indices of contractile function (e.g., LVDP, RPP, +dP/dt, and −dP/dt) were normalized to baseline values and plotted as a function of time in reperfusion in the hearts from intact and GDX mice (Fig. 9). Data were analyzed with a mixed two-way ANOVA with time and GDX as main factors. The detailed statistical analysis is presented in Additional file 3: Table S3. Results showed that LVDP recovered significantly better in the GDX mouse hearts compared to the hearts from age-matched intact males at all time points throughout reperfusion (Fig. 9a). Similarly, RPP, +dP/dt, and −dP/dt recovered substantially better in the hearts from GDX mice compared to intact mice (Fig. 9b, c, and d). We also compared coronary flow rates and reperfusion contractures in the hearts from intact and GDX mice. Coronary flow rates were similar in both groups (Fig. 10a), which suggests that alterations in myocardial perfusion do not explain improved recovery of function in the GDX male hearts. However, reperfusion contractures were markedly attenuated by GDX (Fig. 10b), consistent with reduced myocardial injury in reperfusion in the GDX animals. Together, these results demonstrate that the hearts from young GDX mice with very low testosterone levels exhibited much better functional recovery and less myocardial injury after cardioplegia followed by I/R when compared to the hearts from young mice with normal testosterone levels.

## Discussion

The overall goals of this study were to determine sex- and age-specific differences in the recovery of the hearts treated with STH2 cardioplegia and to explore potential mechanisms involved in differences in recovery. Cardiac contractions recovered equally in reperfusion in the female hearts, regardless of age, and there was no difference in infarct areas. By contrast, the hearts from old males had smaller infarcts and superior recovery of contractions in reperfusion compared to young males. There was also a sex-specific difference in the young group, where young males had larger infarcts and worse functional recovery than young females. Additional experiments using young male GDX mice were done to elucidate the possible beneficial role of low testosterone levels in protecting the heart against I/R injury following cardioplegic arrest. Interestingly, the hearts from GDX males exhibited much better recovery of function and smaller infarct areas than age-matched intact males. Together, these findings suggest that low serum testosterone levels in older males may protect the heart against I/R injury.

In the present study, we developed a cardioplegic perfusion model based on common clinical practice, where a surgeon often uses a single dose of hypothermic cardioplegia to protect the heart before cardiac surgery [36]. This is done because clinical studies have shown that a single initial dose of cardioplegia can protect the heart if ischemia does not exceed 90 min [37–39]. Clinical work has also shown that maintenance of hypothermia during cardioplegia is also important, as it is associated with lower O_2_ demand and reduced basal energy requirement of the myocardium [32]. Hence, the present study used a cardioplegia protocol with a single dose of ice-cold solution, then 90 min of room temperature ischemia followed by reperfusion. This protocol was designed to maximally protect the heart against I/R injury. Variations in the recovery of the heart function in reperfusion in different experimental groups suggest that there are sex- and age-specific differences in the benefits of STH2 cardioplegic treatment.

While one might assume that aging is associated with increased myocardial susceptibility to I/R injury, this is not, in fact, seen in all studies [22, 40]. For instance, one study done in male rats reported that the severity of myocardial damage following I/R insult was actually substantially higher in 16-month-old rats compared to 24-month-old animals [40]. They concluded that 16-month-old rats had a lower ability to eliminate hydrogen peroxide compared to 24-month-old rats, causing over-production of oxygen-free radicals and cardiomyocyte damage [40]. Consistent with these findings in a classic I/R model, we found that the recovery of cardiac function in the hearts protected with cardioplegia was substantially better in old males when compared to younger males. Infarcts were also markedly smaller in older males when compared to young adult males. Interestingly, studies in humans have shown comparable outcomes following coronary artery bypass surgery in individuals over the age of 80 years when compared to younger patients [22]. It is important to note, however, that most of these octogenarian patients were female [22], consistent with our finding that females showed no age-associated difference in either infarct size or recovery of function during reperfusion following cardioplegic arrest.

We explored potential mechanisms underlying the smaller infarcts and improved recovery of function in old males. Some previous studies have shown that testosterone has cardioprotective effects in the setting of ischemia [41, 42], although this is controversial [43–48]. Here, we revealed a potential protective role of low testosterone in reducing infarct size and enhancing cardiac recovery after cardioplegic arrest. We found that serum testosterone concentrations declined markedly with age in males, which suggested that low testosterone levels might have protected the aging heart against I/R injury. In support of this, bilateral GDX of male mice mimicked the beneficial effects of age on infarct area and contractile recovery in our cardioplegia model. Since the heart can accumulate testosterone at higher concentrations than other androgen target organs, its role in cardiac injury may be important [49].

Activation of p38 MAPK is reduced in the hearts from castrated male rats subjected to global I/R injury compared to intact males [50, 51]. As activation of p38 MAPK promotes inflammatory cytokine production and apoptosis in cardiomyocytes, this may explain why the hearts from intact males are more susceptible to I/R injury than the hearts from castrated males [50, 51]. In support of this, low testosterone levels reduce proinflammatory cytokine production (TNF-α, IL-1β, and IL-6) in young rats (3 months) that have either been castrated or treated with the androgen receptor antagonist flutamide [50]. In addition, lower expression of apoptosis-related proteins (caspase-3 and caspase-11) and higher levels of the antiapoptotic protein, Bcl-2, have been reported in young rats with low serum testosterone levels when compared to controls [42, 49]. Alternatively, Huang and colleagues [52] showed that recovery of myocardial function after I/R injury was much worse in young males compared to age-matched females, castrated males, or flutamide-treated males. They and others suggest that cardiac ischemic injury is mediated through testosterone-induced downregulation of the Akt pathway in the young male hearts [52, 53]. In contrast, estrogen is believed to activate Akt pathway in females and thus inhibit myocyte apoptosis during I/R [54]. Whether these mechanisms underlie beneficial effects of low testosterone on functional outcomes after cardioplegic arrest and reperfusion is unclear, and additional studies are now warranted.

It is clear that estrogen levels can affect the response to myocardial I/R injury [55]. For example, it is well established that I/R injury is exacerbated by ovariectomy in adult female rats and this can be attenuated by estrogen [56, 57] or by testosterone plus estrogen [58]. It is also known that the accumulation of adipose tissue increases the levels of aromatase [59, 60], a key enzyme required for the biosynthesis of estrogen from testosterone [60, 61]. Thus, it is possible that age-related increase in adipose tissue could increase levels of circulating estradiol in aging animals in the present study. When we quantified estradiol levels in young adult and aged mice of both sexes, we found that estradiol levels were highest in young adult females, but were also detectable in older males. However, estradiol levels were below limits of detection in young males, older females, and in GDX mice. Thus, while higher levels of estrogen may contribute to cardioprotection in the young females and older males, this is unlikely to explain the cardioprotection we observed in GDX mice and in older females.

Our study also showed that there was a sex difference in responses to cardioplegic arrest followed by I/R in the young group. We found that functional recovery was worse in the young males than in the young females and that young males had larger infarcts compared to age-matched females. Previous studies in a variety of preclinical models have shown that the young adult female hearts are more resistant to ischemic injury than the young adult male hearts [7, 48]. Our work extends these findings to show that this female advantage is also seen when the hearts are arrested with a cardioplegic solution designed specifically to protect the heart during cardiac surgery.

Despite developing an experimental protocol that is similar to the clinical setting of cardiac surgery, our study does have limitations. Cardiac surgery is performed in patients with diseased hearts, whereas here, the hearts came from mice with no known cardiovascular disease. In addition, we were not able to examine heart function at later, more clinically relevant post-ischemic time points (e.g., 12 or 24 h post-reperfusion). Further experiments could explore age- and sex-dependent effects on these endpoints in in vivo models of cardioplegic arrest and reperfusion.

## Conclusions

This study developed an animal model of cardioplegia and demonstrated that testosterone deficiency plays a role in protecting the older male heart from I/R injury following cardioplegic arrest. In contrast to males, the female hearts were equally protected from I/R injury by STH2 cardioplegia regardless of age. Overall, this study suggests that high testosterone levels in younger men may contribute to worse outcomes following cardiac surgery.

### Perspectives and significance

Our findings strongly suggest that higher testosterone levels in men contribute to worse outcomes following cardioplegic arrest and reperfusion during cardiac surgery. However, these negative outcomes in younger men do not necessarily forecast poor outcomes in older men. Our data suggest that older men with lower testosterone levels may have better recovery of function after cardioplegia and I/R in the setting of cardiac surgery. These results also suggest that testosterone supplementation may be unadvisable in individuals of all ages prior to cardiac surgery, an idea that is motivating our further inquiries.

## Additional files

Additional file 1: Two-way ANOVA for morphology, infarct size, and testosterone in young and aged male and female mice. (DOCX 15 kb)

Additional file 2: Three-way mixed ANOVA of Langendorff functional data in young and aged male and female mice. (DOCX 16 kb)

Additional file 3: Two-way mixed ANOVA of functional data for intact vs GDX. (DOCX 15 kb)

## Data Availability

All data are available from the corresponding author upon request.
