# Supplementary material for: Ischemia and reperfusion injury following cardioplegic arrest is attenuated by age and testosterone deficiency in male but not female mice
Source: Biol Sex Differ. 2019 Aug 23;10:42. doi: 10.1186/s13293-019-0256-4 (PMC6708213; doi:10.1186/s13293-019-0256-4)
Supplement: Supplementary file 1 — Two-way ANOVA for morphology, infarct size, and testosterone in young and aged male and female mice. (DOCX 15 kb) [file 13293_2019_256_MOESM1_ESM.docx]

**Additional file 1: Table S1. Two-way ANOVA for morphology, infarct size and testosterone in young and aged male and female mice**

| Factor^a^ | DF | Eta Squared | F(DFn,DFd)t | p value |
| --- | --- | --- | --- | --- |
| **Heart weight** | | | | |
| Between subject effects |  |  |  |  |
| Sex | 1 | 0.307 | F(1,19)=8.414 | p=0.009 |
| Simple main effect of sex |  |  |  |  |
| Old | 1 | 0.318 | (F1,19)=8.855 | p=0.009 |
| **HW:Tibia Length** |  |  |  |  |
| Between subject effects |  |  |  |  |
| Sex | 1 | 0.354 | F(1,19)=10.412 | p=0.004 |
| Simple main effect of age |  |  |  |  |
| Male | 1 | 0.192 | (F1,19)=4.526 | p=0.047 |
| Simple main effect of sex |  |  |  |  |
| Old | 1 | 0.371 | (F1,19)=11.213 | p=0.003 |
| **Infarct size** | | | | |
| Between subject effects |  |  |  |  |
| Age | 1 | 0.594 | F(1,15)=21.929 | p<0.0005 |
| Sex*Age | 1 | 0.430 | F(1,15)=11.317 | p=0.004 |
| Simple main effect of Age |  |  |  |  |
| Male | 1 | 0.692 | F(1,15)=33.672 | p<0.0005 |
| Simple main effect of Sex |  |  |  |  |
| Young | 1 | 0.371 | F(1,15)=8.849 | p=0.009 |
| **Testosterone Concentration** | | | | |
| Between subject effects |  |  |  |  |
| Sex | 1 | 0.640 | F(1,12)=21.367 | p=0.001 |
| Age | 1 | 0.436 | F(1,12)=9.270 | p=0.010 |
| Sex*Age | 1 | 0.370 | F(1,12)=7.061 | p=0.021 |
| Simple main effect of Age |  |  |  |  |
| Male | 1 | 0.583 | F(1,12)=16.797 | p=0.001 |
| Simple main effect of Sex |  |  |  |  |
| Young | 1 | 0.662 | F(1,12)=23.469 | p<0.0005 |

^a^ Results of two way ANOVA with two main factors (age, sex).
