# Supplementary material for: Ischemia and reperfusion injury following cardioplegic arrest is attenuated by age and testosterone deficiency in male but not female mice
Source: Biol Sex Differ. 2019 Aug 23;10:42. doi: 10.1186/s13293-019-0256-4 (PMC6708213; doi:10.1186/s13293-019-0256-4)
Supplement: Supplementary file 2 — Three-way mixed ANOVA of Langendorff functional data in young and aged male and female mice. (DOCX 16 kb) [file 13293_2019_256_MOESM2_ESM.docx]

**Additional file 2: Table 2. Three way mixed ANOVA of Langendorff functional data in young and aged male and female mice**

| Factor^a^ | DF | Eta Squared | F(DFn,DFd)t | p value |
| --- | --- | --- | --- | --- |
| **LVDP (%)** |  |  |  |  |
| Within subject effects |  |  |  |  |
| Time | 2.113 | 0.775 | F(2.113,38.041)=61.995 | P<0.0005 |
| Time*age | 2.113 | 0.184 | F(2.113,38.041)=4.055 | P=0.023 |
| Time*age*sex - main 3 way interaction | 2.113 | 0.181 | F(2.113,38.041)=3.985 | p=0.025 |
| Between subject effect |  |  |  |  |
| Age | 1 | 0.204 | F(1,18)=4.611 | P=0.046 |
| Simple two way interactions of sex*age | | | | |
| Time 20 | 1 |  | F(1,18)=5.002 | P=0.038 |
| Time 25 | 1 |  | F(1,18)=4.972 | P=0.039 |
| Time 30 | 1 |  | F(1,18)=4.852 | P=0.041 |
| Simple simple main effect of sex, young | | | | |
| Time 20 | 1 |  | F(1,18)=4.654 | P=0.045 |
| Time 25 | 1 |  | F(1,18)=4.774 | P=0.042 |
| Time 30 | 1 |  | F(1,18)=4.752 | P=0.043 |
| Simple simple main effect of age, male | | | | |
| Time 15 | 1 |  | F(1,18)=5.932 | P=0.025 |
| Time 20 | 1 |  | F(1,18)=10.004 | P=0.005 |
| Time 25 | 1 |  | F(1,18)=10.902 | P=0.004 |
| Time 30 | 1 |  | F(1,18)=11.818 | P=0.003 |
| **RPP (%)** |  |  |  |  |
| Within subject effects |  |  |  |  |
| Time | 2.034 | 0.728 | F(2.304,36.615)=48.063 | P<0.0005 |
| Time*age | 2.034 | 0.176 | F(2.304,36.615)=3.843 | P=0.030 |
| Simple simple main effect of age, male | | | | |
| Time 15 | 1 |  | F(1,18)=4.506 | P=0.048 |
| Time 20 | 1 |  | F(1,18)=8.038 | P=0.011 |
| Time 25 | 1 |  | F(1,18)=7.065 | P=0.016 |
| Time 30 | 1 |  | F(1,18)=8.304 | P=0.010 |
| **+dp/dt (%)** |  |  |  |  |
| Within subject effects |  |  |  |  |
| Time | 2.104 | 0.776 | F(2.104,37.879)=62.210 | P<0.0005 |
| Time*age | 2.104 | 0.182 | F(2.104,37.879)=4.017 | P=0.024 |
| Time*age*sex - main 3 way interaction | 2.104 | 0.153 | F(2.104,37.879)=3.249 | p=0.048 |
| Between subject effect |  |  |  |  |
| Age | 1 | 0.201 | F(1,18)=4.533 | P=0.047 |
| Simple simple main effect of sex, young | | | | |
| Time 25 | 1 |  | F(1,18)=4.740 | P=0.043 |
| Time 30 | 1 |  | F(1,18)=4.429 | P=0.050 |
| Simple simple main effect of age, male | | | | |
| Time 15 | 1 |  | F(1,18)=5.367 | P=0.033 |
| Time 20 | 1 |  | F(1,18)=9.086 | P=0.007 |
| Time 25 | 1 |  | F(1,18)=9.585 | P=0.006 |
| Time 30 | 1 |  | F(1,18)=10.138 | P=0.005 |
| **-dp/dt (%)** |  |  |  |  |
| Within subject effects |  |  |  |  |
| Time | 2.048 | 0.772 | F(2.048,36.856)=61.002 | P<0.0005 |
| Time*age | 2.048 | 0.163 | F(2.048,36.856)=3.510 | P=0.039 |
| Simple simple main effect of age, male | | | | |
| Time 15 | 1 |  | F(1,18)=4.727 | P=0.043 |
| Time 20 | 1 |  | F(1,18)=7.716 | P=0.012 |
| Time 25 | 1 |  | F(1,18)=8.032 | P=0.011 |
| Time 30 | 1 |  | F(1,18)=8.774 | P=0.008 |

^a^ Results of three way mixed ANOVA with two between factors (age, sex) and one within factor (time).
