# Supplementary material for: Ischemia and reperfusion injury following cardioplegic arrest is attenuated by age and testosterone deficiency in male but not female mice
Source: Biol Sex Differ. 2019 Aug 23;10:42. doi: 10.1186/s13293-019-0256-4 (PMC6708213; doi:10.1186/s13293-019-0256-4)
Supplement: Supplementary file 3 — Two-way mixed ANOVA of functional data for intact vs GDX. (DOCX 15 kb) [file 13293_2019_256_MOESM3_ESM.docx]

**Additional file 3: Table 3. Two way mixed ANOVA of functional data for intact vs GDX**

| Factor^a^ | DF | Eta Squared | F(DFn,DFd)t | P value |
| --- | --- | --- | --- | --- |
| **LVDP (%)** | | | | |
| Within subject effects |  |  |  |  |
| Time | 2.062 | 0.795 | F(2.062,14.434)=27.170 | p<0.0005 |
| Time*GDX | 2.062 | 0.633 | F(2.062,14.434)=12.080 | p=0.001 |
| Between subject effects |  |  |  |  |
| GDX | 1 | 0.895 | F(1,7)=24.033 | p=0.002 |
| Time 5 | 1 | 0.744 | F(1,7)=20.308 | p=0.003 |
| Time 10 | 1 | 0.878 | F(1,7)=50.279 | p<0.0005 |
| Time 15 | 1 | 0.783 | F(1,7)=25.243 | p=0.002 |
| Time 20 | 1 | 0.758 | F(1,7)=21.912 | p=0.002 |
| Time 25 | 1 | 0.675 | F(1,7)=14.555 | p=0.007 |
| Time 30 | 1 | 0.629 | F(1,7)=11.850 | p=0.011 |
| **RPP (%)** | | | | |
| Within subject effects |  |  |  |  |
| Time | 2.037 | 0.811 | F(2.037,14.261)=30.022 | p<0.0005 |
| Time*GDX | 2.037 | 0.648 | F(2.037,14.261)=12.880 | p=0.001 |
| Between subject effects |  |  |  |  |
| GDX | 1 | 0.765 | F(1,7)=22.828 | p=0.002 |
| Time 5 | 1 | 0.785 | F(1,7)=25.617 | p=0.001 |
| Time 10 | 1 | 0.802 | F(1,7)=28.443 | p=0.001 |
| Time 15 | 1 | 0.771 | F(1,7)=23.600 | p=0.002 |
| Time 20 | 1 | 0.754 | F(1,7)=21.477 | p=0.002 |
| Time 25 | 1 | 0.683 | F(1,7)=15.051 | p=0.006 |
| Time 30 | 1 | 0.651 | F(1,7)=13.039 | p=0.009 |
| **+dP/dt (%)** | | | | |
| Within subject effects |  |  |  |  |
| Time | 2.092 | 0.791 | F(2.092,14.646)=26.486 | p<0.0005 |
| Time*GDX | 2.037 | 0.648 | F(2.092,14.646)=12.123 | p=0.001 |
| Between subject effects |  |  |  |  |
| GDX | 1 | 0.769 | F(1,7)=23.262 | p=0.002 |
| Time 5 | 1 | 0.743 | F(1,7)=20.211 | p=0.003 |
| Time 10 | 1 | 0.864 | F(1,7)=44.471 | p<0.0005 |
| Time 15 | 1 | 0.778 | F(1,7)=24.497 | p=0.002 |
| Time 20 | 1 | 0.759 | F(1,7)=22.104 | p=0.002 |
| Time 25 | 1 | 0.674 | F(1,7)=14.500 | p=0.007 |
| Time 30 | 1 | 0.622 | F(1,7)=11.503 | p=0.012 |
| **dP/dt (%)** | | | | |
| Within subject effects |  |  |  |  |
| Time | 2.116 | 0.788 | F(2.116,14.812)=25.980 | p<0.0005 |
| Time*GDX | 2.116 | 0.622 | F(2.116,14.812)=11.518 | p=0.001 |
| Between subject effects |  |  |  |  |
| GDX | 1 | 0.771 | F(1,7)=23.517 | p=0.002 |
| Time 5 | 1 | 0.755 | F(1,7)=21.687 | p=0.002 |
| Time 10 | 1 | 0.868 | F(1,7)=46.116 | p<0.0005 |
| Time 15 | 1 | 0.777 | F(1,7)=24.447 | p=0.002 |
| Time 20 | 1 | 0.758 | F(1,7)=21.878 | p=0.002 |
| Time 25 | 1 | 0.661 | F(1,7)=13.663 | p=0.008 |
| Time 30 | 1 | 0.600 | F(1,7)=10.483 | p=0.014 |

^a^ Results of two way mixed ANOVA with GDX and time as main factors.
